# Exploring the Impact of Vitamin D Supplementation on Metabolic Syndrome Variables in Postmenopausal Women: A Comprehensive Review

**DOI:** 10.7759/cureus.61806

**Published:** 2024-06-06

**Authors:** Shivani Singh, Neema Acharya, Sourya Acharya, Megha Karnik, Aishwarya Beedkar, Dharmesh Patel

**Affiliations:** 1 Obstetrics and Gynecology, Jawaharlal Nehru Medical College, Datta Meghe Institute of Higher Education and Research, Wardha, IND; 2 Obstetrics and Gynaecology, Jawaharlal Nehru Medical College, Datta Meghe Institute of Higher Education and Research, Wardha, IND; 3 Medicine, Jawaharlal Nehru Medical College, Datta Meghe Institute of Higher Education and Research, Wardha, IND

**Keywords:** inflammation markers, lipid profiles, insulin sensitivity, vitamin d supplementation, postmenopausal women, metabolic syndrome

## Abstract

Metabolic syndrome poses a significant health concern, particularly among postmenopausal women who are vulnerable to its adverse effects. Emerging evidence suggests a potential role of vitamin D in mitigating metabolic syndrome risk factors, prompting interest in its supplementation as a therapeutic intervention. This comprehensive review examines the impact of vitamin D supplementation on metabolic syndrome variables in postmenopausal women. Through a systematic synthesis of existing literature, we assess the evidence supporting the beneficial effects of vitamin D on insulin sensitivity, lipid profiles, and inflammation markers in this population. While findings suggest potential benefits, uncertainties remain regarding optimal dosage and duration of supplementation. Implications for clinical practice underscore the importance of assessing vitamin D status and considering supplementation as part of a comprehensive approach to metabolic health management. Furthermore, public health initiatives promoting adequate vitamin D intake may help mitigate the prevalence of metabolic syndrome and associated complications. However, further research is warranted to elucidate the underlying mechanisms, establish optimal supplementation protocols, and explore potential interactions with other nutrients or medications. Long-term randomized controlled trials are needed to evaluate the sustained effects of vitamin D supplementation on metabolic health outcomes in postmenopausal women.

## Introduction and background

Metabolic syndrome (MetS) refers to a cluster of conditions that occur together, increasing the risk of heart disease, stroke, and type 2 diabetes. These conditions include abdominal obesity, high blood pressure, elevated blood sugar levels, and abnormal cholesterol or triglyceride levels [1]. Postmenopausal women are a particularly vulnerable population when it comes to metabolic health. The hormonal changes that occur during menopause, including decreases in estrogen levels, can lead to alterations in metabolism and an increased risk of developing MetS [2].

Vitamin D, often called the "sunshine vitamin," is crucial to bone health. However, emerging evidence suggests that vitamin D may also affect metabolic health. It has been implicated in various physiological processes, including insulin sensitivity, inflammation, and lipid metabolism [3]. This comprehensive review aims to explore the impact of vitamin D supplementation on MetS variables in postmenopausal women. By synthesizing existing literature and examining the findings of relevant studies, we aim to elucidate the potential benefits or limitations of vitamin D supplementation in improving metabolic health outcomes in this vulnerable population.

## Review

Metabolic syndrome in postmenopausal women

Prevalence and Risk Factors

The prevalence of MetS varies across different populations, with rates ranging from 13.8% in premenopausal women to as high as 60% in postmenopausal women [4,5]. Research indicates that menopause is a significant risk factor for cardiometabolic diseases, including MetS, type 2 diabetes, and cardiovascular diseases [6]. Postmenopausal women face an elevated risk of developing MetS due to factors such as hormonal changes, weight gain, and reduced physical activity [5]. Studies have shown that the prevalence of MetS can vary depending on the diagnostic criteria used. For instance, employing the NCEP ATP III criterion, the prevalence of MetS was higher among postmenopausal women than premenopausal women in several studies [4]. Furthermore, age plays a pivotal role in the prevalence of MetS among women, with postmenopausal women exhibiting higher age-specific prevalence compared to premenopausal women [4]. Regarding risk factors for metabolic disease following menopause, the research underscores the importance of midlife exercise and a healthy diet in mitigating the risks associated with MetS and other related conditions [7]. The transition to menopause and the postmenopausal period are considered vulnerable periods for the development of MetS due to declining estrogen levels and an increased risk of insulin resistance [8].

Health Consequences and Implications

Menopause stands out as a significant factor contributing to various health ramifications, particularly those related to metabolic disorders. Postmenopausal women face an elevated risk of developing cardiometabolic diseases, such as MetS, type 2 diabetes, and cardiovascular diseases [6]. The onset of MetS is intricately linked to menopause, exerting an impact on lipid profiles and other metabolic parameters [9]. The research underscores the heightened vulnerability of women to developing MetS following menopause, underscoring the importance of midlife exercise and a balanced diet in mitigating these risks [7]. Furthermore, studies have established a correlation between vitamin D deficiency and MetS, with insufficient levels of vitamin D being associated with elevated fasting blood glucose, blood pressure, and triglycerides in individuals with MetS [10]. A comprehensive understanding of the implications of menopause on metabolic health is imperative for the development of effective preventive strategies and interventions aimed at reducing the risks associated with metabolic disorders among postmenopausal women. Further exploration and investigation in this realm are essential to advance our understanding and enhance health outcomes for this population.

Vitamin D and metabolic health

Functions of Vitamin D in the Body

**Figure 1:**
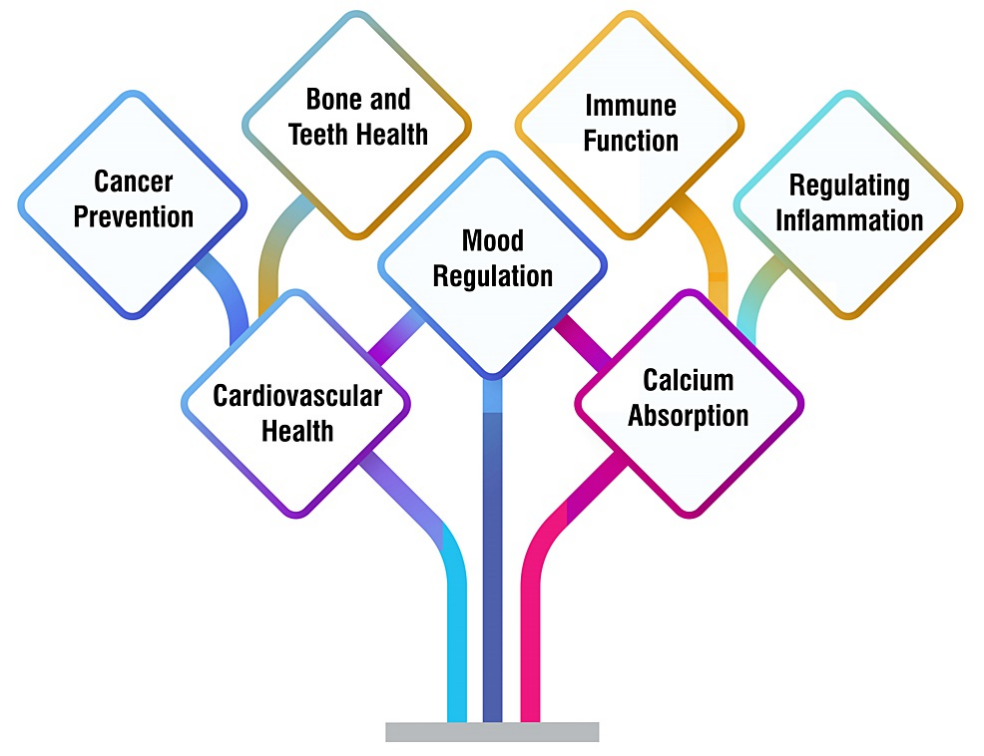
Functions of vitamin D in the body Image credit: Bhushan Wandile

Vitamin D plays a crucial role in various aspects of health, including bone and teeth health. Inadequate vitamin D levels can result in conditions such as rickets in children characterized by bone softening and dental issues. In adults, a deficiency in vitamin D can impact bone health and contribute to conditions such as osteoporosis [11,12]. Adequate vitamin D intake supports immune function and reduces the risk of autoimmune diseases. Vitamin D regulates immune responses and may help lower the risk of infections and autoimmune conditions [13]. Furthermore, vitamin D is involved in regulating inflammation in the body. Optimal vitamin D levels may help modulate inflammatory responses, potentially reducing the risk of chronic inflammatory conditions [12]. One of the primary functions of vitamin D is to regulate the absorption of calcium and phosphorus in the body. This is crucial for maintaining strong bones and teeth and supporting various physiological processes that rely on these minerals [12]. Research suggests a potential role of vitamin D in promoting cardiovascular health. Adequate vitamin D levels are associated with a reduced risk of heart diseases such as hypertension, heart failure, and stroke. However, further studies are needed to understand this relationship fully [12]. Some studies indicate that vitamin D may influence mood regulation and mental health. Low levels of vitamin D have been linked to an increased risk of depression, and supplementation may help improve mood in individuals with deficiencies [11]. While the evidence is mixed, some studies suggest that adequate levels of vitamin D may lower the risk of certain cancers such as breast, colon, or rectal cancer. However, more research is needed to clarify the role of vitamin D in cancer prevention [11]. Functions of vitamin D in the body are shown in Figure 1.

Evidence Linking Vitamin D Deficiency to Metabolic Syndrome

Vitamin D deficiency has been linked to MetS, a constellation of metabolic and vascular disorders that elevate the risk of cardiovascular disease and diabetes. Studies have demonstrated an inverse association between plasma vitamin D concentrations and the defining features of MetS, including elevated serum levels of glucose, total cholesterol, low-density lipoproteins, triglycerides, glycosylated hemoglobin, and a high body mass index (BMI) [14]. Numerous investigations have detailed the advantageous effects of vitamin D supplementation in enhancing outcomes among individuals with MetS [14]. A cross-sectional study conducted at a tertiary care hospital in northern India revealed a prevalent vitamin D deficiency (<20 ng/mL) among patients diagnosed with MetS. Moreover, the study uncovered a significant negative correlation between vitamin D levels and various components of MetS, such as diastolic blood pressure, fasting blood glucose, total cholesterol, triglyceride levels, and low-density lipoprotein levels [15]. The collective evidence strongly suggests an association between vitamin D deficiency and MetS, along with its risk factors, underscoring the imperative for further research to elucidate the underlying mechanisms and potential benefits of vitamin D supplementation in the prevention and management of MetS [15].

Impact of vitamin D supplementation

Studies Assessing the Effect of Vitamin D Supplementation on Metabolic Syndrome Variables

Several studies have highlighted an inverse relationship between plasma vitamin D concentrations and the defining features of MetS. These features include elevated serum levels of glucose, total cholesterol, low-density lipoproteins, triglycerides, glycosylated hemoglobin, and a high BMI [14,16,17]. This suggests that higher vitamin D levels may be associated with a reduced risk of developing MetS. Furthermore, research has indicated that vitamin D supplementation may benefit adults with MetS by improving various outcomes related to health. These benefits include improvements in anthropometric measurements, blood pressure, blood lipid profile, glycemia, oxidative stress, and vitamin D toxicity [14,18]. These findings suggest that vitamin D supplementation could be a potential intervention for managing MetS and its associated complications. However, controversies exist regarding the effectiveness of vitamin D supplementation in treating MetS. While some studies demonstrate positive effects on variables such as insulin resistance and hypertension, the overall impact on blood lipid profiles still needs to be more conclusive [18]. Thus, further research is warranted to clarify the effectiveness of vitamin D supplementation in preventing or treating MetS. This includes well-designed clinical trials with larger sample sizes and more extended follow-up periods to assess the long-term effects of supplementation on metabolic health outcomes.

Dosage and Duration Considerations

Dosage and duration considerations for vitamin D supplementation in postmenopausal women should be tailored to individual needs and serum 25-hydroxy-vitamin D (25OH-D) levels. The recommended daily vitamin D intake is 400 international units (IU) for children up to 12 months old, 600 IU for individuals aged 1 to 70 years, and 800 IU for those over 70 years [19]. However, these guidelines may only be suitable for some, and serum 25OH-D levels should be considered when determining the appropriate dose of vitamin D supplementation. For adults diagnosed with vitamin D deficiency (serum 25OH-D levels below 30 nmol/L), the recommended dosage is 50,000 IU (1 capsule) once weekly [20]. In cases where serum 25OH-D levels fall between 30 and 50 nmol/L, a weekly dose of 50,000 IU (1 capsule) may suffice [20]. Similarly, if serum 25OH-D levels range between 50 and 124 nmol/L, a weekly dose of 50,000 IU (1 capsule) may be appropriate [20]. However, it is crucial to monitor serum 25OH-D levels following the initiation of vitamin D supplementation to ensure adequate vitamin D status. The duration of vitamin D supplementation should be individualized based on the initial serum 25OH-D levels and the desired target levels. Vitamin D supplementation should be continued until the desired serum 25OH-D levels are attained and maintained. Regularly monitoring serum 25OH-D levels ensures the supplementation dose remains appropriate and effective.

Mechanisms Underlying the Observed Effects

Vitamin D undergoes a complex process of transport and metabolism within the body. It is carried through the bloodstream by vitamin D binding protein to the liver, where it undergoes various metabolic transformations. These metabolic processes significantly influence vitamin D levels and the metabolites that circulate, ultimately impacting its physiological functions [21]. Numerous studies have highlighted a compelling link between vitamin D deficiency and MetS, along with its associated outcomes, such as type 2 diabetes mellitus [15]. This association underscores the critical role of adequate vitamin D levels in managing metabolic health. Ensuring sufficient vitamin D status may be a preventive measure against the development and progression of MetS and related metabolic disorders. Furthermore, research suggests that the response to vitamin D supplementation may vary among individuals, particularly regarding BMI. Individuals with a higher BMI may respond differently to vitamin D supplementation. Specifically, supplementation with vitamin D has been associated with increased serum vitamin D-related biomarkers. However, this response appears to be blunted in participants with overweight or obesity at baseline [22]. These findings highlight the need for tailored approaches to vitamin D supplementation, considering individual characteristics such as BMI, to optimize its effectiveness in improving metabolic health outcomes.

Potential confounding factors and limitations

Dietary and Lifestyle Factors

Dietary and lifestyle factors are pivotal in developing and managing MetS among individuals. Numerous studies have shed light on the significant impact of lifestyle modifications on MetS risk factors, underscoring the importance of interventions that target diet and physical activity [23,24]. Research findings consistently highlight lifestyle risk factors such as poor dietary habits, physical inactivity, smoking, excessive alcohol consumption, and sedentary behavior as contributors to the heightened risk of MetS [23]. Additionally, factors including obesity, insulin resistance, aging, hormonal imbalances, and abdominal obesity have been closely associated with MetS and are susceptible to modulation through dietary and lifestyle adjustments [24,25]. Furthermore, studies have demonstrated the efficacy of various dietary interventions in mitigating MetS risk factors. These interventions encompass alterations in macronutrient composition, use of meal replacements, adoption of low-fat, high-carbohydrate diets supplemented with long-chain n-3 polyunsaturated fatty acids, and implementation of lifestyle changes such as weight reduction and regular physical activity [24]. Moreover, the adoption of a healthy lifestyle score, incorporating factors such as smoking cessation, maintenance of a healthy BMI, engagement in regular physical activity, and adherence to a nutritious diet abundant in fruits, vegetables, legumes, and whole grains, has been associated with a notable reduction in the risk of MetS [26].

Variability in Study Designs and Populations

Ethnic Differences and Vitamin D Requirements: It is imperative to acknowledge the higher vitamin D requirements in obese individuals and tailor interventions accordingly to achieve optimal 25(OH)D concentrations through vitamin D dosing adaptations during trials. Additionally, potential ethnic disparities should be considered when determining vitamin D supplementation strategies [27]. Ethnicity can significantly influence vitamin D status due to variations in skin pigmentation, sunlight exposure, dietary habits, and genetic factors, highlighting the importance of addressing these differences in study designs and interventions.

Design Considerations: Accurate and standardized measurements of vitamin D status are paramount for the success of future trials investigating its impact on metabolic health. Researchers must account for seasonal variations in vitamin D levels, different sources of vitamin D (dietary intake, sunlight exposure, supplementation), and bioavailability when designing studies [27]. These factors can significantly affect the interpretation of study findings and must be carefully controlled to ensure the reliability and validity of the results.

Confounding Factors: While observational studies have consistently linked low vitamin D status to an increased risk of various diseases, randomized controlled trials (RCTs) face challenges such as low response rates and biases that may impact their validity and generalizability [28]. Biases, such as the recruitment of individuals already sufficient in vitamin D, can limit the ability to detect the beneficial effects of supplementation. Therefore, efforts must be made to mitigate these confounding factors through rigorous study design and statistical analysis.

Dose-Response Relationship: Understanding the dose-response relationship between vitamin D intake and serum 25(OH)D concentrations is critical for optimizing supplementation strategies. Different dosing regimens (daily, weekly, or monthly) may yield similar serum 25(OH)D levels, and the average nutritional intake of vitamin D in the general population often falls below recommended levels [28]. Therefore, establishing effective dosing regimens that ensure sufficient vitamin D intake is essential for improving metabolic health outcomes.

Optimal Strategies for Supplementation: While a daily supplemental dose of 20 µg (800 IU) of vitamin D is generally considered sufficient for most individuals to achieve adequate serum levels, upcoming large RCTs may provide valuable insights into the safety and efficacy of higher doses [28]. Addressing these factors and limitations in study designs and populations is crucial for accurately evaluating the impact of vitamin D supplementation on MetS variables in postmenopausal women. Further research using personalized approaches and focusing on specific populations may help elucidate the role of vitamin D in effectively managing MetS [29].

Challenges in Interpreting Results

Interpreting findings from studies investigating the impact of vitamin D supplementation on MetS variables in postmenopausal women poses several challenges due to various factors. While RCTs are typically regarded as the gold standard for research, they come with limitations that may compromise their validity. These limitations include low response rates, biases affecting internal validity (such as the recruitment of individuals already sufficient in vitamin D), and difficulties in studying long-term outcomes requiring extended participation, compliance, and retention [30]. Furthermore, large-scale vitamin D RCTs have predominantly targeted the general population rather than specific subgroups vulnerable to vitamin D deficiency, potentially diluting the observed effects seen in observational studies [27]. The dose-response relationship between vitamin D intake and serum 25(OH)D concentrations is not linear, necessitating careful consideration in study design and result interpretation [28]. Vitamin D supplementation has also demonstrated benefits in certain health outcomes, such as preventing acute respiratory infections and cancer, but these effects are typically modest. They may vary based on individual characteristics [28]. To address these challenges and advance our understanding of the effects of vitamin D supplementation, future research should prioritize personalized approaches that target populations susceptible to vitamin D deficiency. Factors such as baseline vitamin D status, optimal dosing strategies, accurate measurement of vitamin D metabolites, and seasonal variations should be carefully considered in the design and analysis of clinical studies investigating nutrient effects [28]. By tackling these challenges and refining study methodologies, researchers can enhance the reliability and relevance of findings concerning the impact of vitamin D supplementation on MetS variables in postmenopausal women.

## Conclusions

In conclusion, the comprehensive review underscores the potential of vitamin D supplementation to influence MetS variables in postmenopausal women positively. While the evidence suggests beneficial effects on insulin sensitivity, lipid profiles, and inflammation markers, uncertainties remain regarding the optimal dosage and duration of supplementation. These findings carry significant implications for clinical practice, advocating for assessing vitamin D status in at-risk postmenopausal women and considering supplementation as part of a holistic approach to metabolic health management. Moreover, public health initiatives should prioritize promoting adequate vitamin D intake through various means, including diet, sunlight exposure, and supplementation, to mitigate the prevalence of MetS and its associated complications. However, further research is essential to elucidate the underlying mechanisms, establish optimal supplementation protocols, and explore potential interactions with other nutrients or medications. Long-term RCTs are warranted to evaluate the sustained effects of vitamin D supplementation on metabolic health outcomes in this vulnerable population.
